# Exploring the clinical and histopathological characteristics on breast phyllodes tumors predictors and prognosis in a real world

**DOI:** 10.3389/fonc.2025.1550429

**Published:** 2025-04-15

**Authors:** Ye Han, Hong Yu, Congyi Li, Wei Jiang, Huilian Shan

**Affiliations:** ^1^ Breast Oncology Department, Shengjing Hospital Affiliated China Medical University, Shengjing Hospital of China Medical University, Shenyang, Liaoning, China; ^2^ College of Nursing, Dalian Medical University, Dalian, Liaoning, China; ^3^ Pharmacy of Shengjing Hospital Affiliated China Medical University, Shengjing Hospital of China Medical University, Shenyang, Liaoning, China

**Keywords:** wide margin, phyllodes tumor, histology, prognosis, local recurrence

## Abstract

**Study objective:**

Phyllodes tumors of the breast (PT) are rare fibroepithelial tumors with varied clinical and histopathological characteristics, and standardized with wide margins in surgery, a systemic retrospective study of PT could improve our understanding of prognosis.

**Design:**

We conducted a retrospective study spanning 2008-2021, which included 333 cases of PT for chart review. We used logistic regression and comparison tests to evaluate the association between clinical features and local recurrence (LR), as well as to summarize overall survival (OS) and disease-free survival (DFS).

**Setting:**

Phyllodes tumors of the breast exhibit a propensity for a higher recurrence rate. The surgical protocol advocates for achieving wide margins (>1 cm), which presents challenges in clinical practice due to the ambiguity in defining such margins.

**Participants:**

A retrospective screening identified 333 cases of PT for inclusion in the study. Comprehensive data for this analysis was extracted from the clinical patient records.

**Interventions:**

Post-operation, all cases were subjected to a standardized protocol of regular follow-up , with subsequent documentation of follow-up data.

**Main outcome measures:**

At a median follow-up of 79 (inter-quartile range: 28-109) months, recurrence occurred in 9.7% (19/196) of benign, 18.4% (18/98) of borderline, and 28.2% (11/39) of malignant tumors. Local recurrence was not reduced with enlarged margin width (<1 cm vs. >1 cm: odds ratio (OR)=0.84; 95% CI, 0.48 to 1.47; p=0.53), but it was associated with age (<40 vs. >40: OR=2.04; 95% CI, 1.13 to 3.68; p=0.01). LR was significantly correlated with mitosis (<5/HFP vs. >=5/HFP: OR=0.56; 95% CI, 0.32 to 0.98; p=0.003), stromal overgrowth (yes vs. no: OR=0.43; 95% CI, 0.32 to 0.98; p=0.014), and stromal atypia (mild vs. marked: OR=0.59; 95% CI, 0.30 to 1.17; p=0.003).

**Result and conclusion:**

This retrospective study confirmed that recurrence and prognosis were not associated with wide margins in the real world, as suggested by previous guidelines, possibly due to the influence of characteristics such as age, stromal overgrowth, stromal atypia, and mitosis.

## Introduction

Phyllodes tumors (PT) of the breast are rare fibroepithelial neoplasms that comprise approximately 1% of all breast tumors (1). They were named after their distinctive leaf-like histologic architectures, first described in 1838, and exhibit variant histological behaviors spanning from benign, borderline, to malignant subtypes, which are subclassified by the World Health Organization (WHO) since 1981 (2, 3). Histologically, morphological and biological features such as stromal overgrowth, stromal cellularity, stromal atypia, epithelial hyperplasia, tumor necrosis, tumor margins, and mitotic activity per 10 high power field (HPF) count are described to subclassify PT and evaluate the rate of local recurrence (4). The standardized treatment for PT involves surgical removal with wide margins ≥1cm and individually tailored adjuvant therapy, as per National Comprehensive Cancer Network (NCCN) guidelines (5). However, benign phyllodes tumors (BPT) is no longer required for wide margins (≥1cm) since 2022 NCCN guidelines.

The prognosis of PT is conceptually evaluated by local recurrence (LR), overall survival rate (OS) and disease-free survival (DFS) (6). The LR rate of PTs varies from 10% to 40% (average 15%) with distant metastasis reported in approximately 25% of malignant PTs. The 5-year DFS rate is around 80%, considering both LR and distant metastasis, and the 5-year estimated OS and DFS rates are reported to be approximately 85% and 77%, respectively (7, 8). Time to local recurrence has been identified as a predictor of survival in patients with soft tissue sarcoma (9). LR has been reported to be correlated with several independent factors, including positive margins, age, grade, tumor size, cellular atypia, stromal overgrowth, and mitoses (10, 11). However, despite the standardized recommendation for wide margins in surgery, recent debates have cast doubt on the significance of margin width in reducing LR. Some researchers have suggested that there is no significant difference in LR between surgery with narrow margins (<1cm) or wide margins (>1cm) (10).

The ongoing controversy regarding the recommended margins has created a dilemma in the treatment of PT, and further investigation is necessary. Therefore, we conducted a study to collect available data and review the prognosis of PT in our institution to clarify the optimal surgical approach and identify independent risk factors.

## Methods

All the data analyzed in this study were collected from Asian patients treated at Shengjing Hospital of China Medical University between 2008 and 2021, as shown in **Table 1**. Patients who were diagnosed with phyllodes tumors were identified retrospectively from the Hospital Information System (HIS) and classified using the AJCC 8th Edition classification system (12). All clinical characteristics, including age, tumor size, clinical presentation, surgery methods, adjuvant therapy, local recurrences, and systemic metastases, were recorded and analyzed retrospectively. The histological features analyzed in this study included stromal cellularity, stromal overgrowth, cellular atypia, mitosis, borders, heterogeneous component, and focal infiltration.

**Table 1:** Clinicopathologic features of phyllodes tumors patient in the study.

Variables	Benign	Borderline	Malignant	p
	n=196	n=98	n=39	
Age at diagnosis, years, median age	40.5	45	45.5	0.01
Tumor size				
≤5cm	168	62	24	
>5 ≤10cm	21	30	10	0.0013
>10cm	7	6	5	
Laterality				
Left	112	53	17	
Right	83	45	22	0.1454
Double	1	0	0	
Initial operative management				
LE	124	30	5	
WLE	59	34	11	0.0408
BCS	6	8	4	
Mastectomy	7	26	19	
Axillary surgery performed				
SLNB	2	9	11	
ALND	0	3	2	
Number of positive nodes identified				
	0	1	0	
Margin status, INITIAL post-operation				
≤1cm	124	30	5	<0.0001
>1cm	72	68	34	
SECOND operation, n=				
WLE	0	0	0	
Mastectomy	0	1	4	
Recurrence				
No	164	58	29	0.13
Recurred	19	13	1	
Dead	2	0	0	
Lost	11	27	9	
Histopathological Characteristics				
Stromal cellularity				
mild	62	45	1	
moderate	62	39	14	<0.0001
marked	67	9	20	
unknown	0	0	0	
Cellular atypia				
mild	146	50	14	
moderate	40	39	9	<0.0001
marked	10	9	16	
Mitosis				
0-4	194	63	12	
>5 ≤9	2	34	15	<0.0001
>10	0	1	12	
Borders				
pushing	75	85	34	0
infiltrative	0	13	5	
unknown	122	0	0	
Intra-tumoral necrosis				
no	190	89	31	0
yes	6	9	8	
Stromal overgrowth				
no	115	44	10	
yes	81	54	29	0

Surgical treatment for PT included various procedures such as lumpectomy (LE), wide local excision (WLE), breast conserving surgery (BCS), and mastectomy. LE involved the removal of the tumor with a negative margin (width of margin <1cm) through an open incision entrance. On the other hand, WLE involved the removal of the tumor with adjacent tissue (width of margin >1cm). BCS, in contrast, involved the removal of the tumor with pathologic wide margins for malignant tumors. All surgical treatment data were collected according to the medical records.

Patients diagnosed with malignant PT were required to visit the hospital every 3 months after surgery. For benign and borderline PT, patients were advised to have a follow-up visit every 3 to 6 months after resection through outpatient visits or telephone interviews. Breast ultrasound and Mammogram were recommended during the follow-up visit. Follow-up results for all patients were collected through the HIS system and telephonic interviews over the past 13 years. The diagnosis of PT was histologically confirmed and recorded by the Pathologic Department of Shengjing Hospital of China Medical University, including any local recurrence and metastasis.

Raw data of clinical characteristics, surgical treatment, and pathological features were compared using the χ2 test or Fisher’s exact test. The 5-year DFS and OS were presented using Kaplan-Meier curves and analyzed using the log-rank test. Multivariate regression analysis was performed using the Cox proportional hazards model to determine the risk factors for LR. A p-value of <0.05 was considered statistically significant.

## Results

### Clinical characteristics

Between January 2008 and December 2021, a total of 333 cases of PT were screened at Shengjing Hospital of China Medical University. The median age of the patients was 44 years (inter-quartile range, IQR: 34-52 years). The median tumor size was 3cm (IQR:2-4) for benign PT, 4cm (IQR:3-6.75) for borderline PT, and 4cm (IQR:3-8) for malignant PT, with a range of 1cm to 22cm. Among all cases, 58.9% (N=196) were benign PT, 29.4% (N=98) were borderline PT, and 11.7% (N=39) were malignant PT. The median follow-up time was 89.7 months (range: 3-194 months), and all patients were female and diagnosed by the Pathology Department at Shengjing Hospital of China Medical University.

The clinical characteristics of the patients are presented in **Table 1**, stratified by benign PT, borderline PT, and malignant PT. Benign PTs were found to be significantly smaller in size compared to borderline and malignant PTs (p=0.013). The median age of the patients for the three subtypes was 40.5 years for benign PT, 45 years for borderline PT, and 47.8 years for malignant PT, with a significant difference observed (p<0.05). The majority of the PT cases were unilateral, with 54.7% (N=182) occurring in the left breast, 45.0% (N=150) occurring in the right breast, and only 0.3% (N=1) being bilateral.

### Surgery management and margins

In terms of primary management, the most common approach was lumpectomy (47.7%), followed by WLE (31.2%), mastectomy (15.6%), and breast-conserving surgery (5.4%). LE was the initial operative management for 63.3% of benign PT cases, 30.6% of borderline PT cases, and 12.8% of malignant PT cases. Borderline and malignant PT cases were more likely to undergo WLE (34.7% and 28.2%, respectively) and mastectomy (26.5% and 48.7%, respectively) than benign PT cases. Tumor size was classified as T1 in 76.3% of cases (<5cm), T2 in 18.3% of cases (≥5cm and <10cm), and T3 in 5.4% of cases (≥10cm).

Regarding surgical margins, in this study, lumpectomy (LE) was defined as a narrow margin (margin <1 cm), while wide local excision (WLE), breast-conserving surgery (BCS), and mastectomy were defined as wide margins (margin >1 cm). Among patients with benign phyllodes tumors (PT), 124 (63.3%) underwent LE, and 72 (36.7%) underwent surgery with wide margins. In the case of borderline PT, 30 (30.6%) cases underwent LE, and 68 (69.4%) cases were treated with wide margins. For malignant PT, 5 (12.8%) patients underwent LE, and 34 (87.2%) patients underwent surgery with wide margins. However, in this study, the disease-free survival (DFS) and local recurrence rates were not significantly correlated with wide margins, even for malignant PT.

Regarding lymph node evaluation, 6.6% of patients underwent sentinel lymph node biopsy (SLNB), and 1.5% underwent axillary lymph node dissection (ALND), which revealed only one metastatic lymph node. A second operation was required in 1.0% of borderline PT cases and 10.3% of malignant PT cases, and all second operations involved mastectomy. While initial negative margin width was less than 1cm in 68.4% of benign PT cases, 36.7% of borderline PT cases, and 10.3% of malignant PT cases, only 1.5% of women expressed a willingness to undergo a second operation.

### Histopathological characteristics

The study found that mild stromal cellularity was present in 31.6% (62/196) of benign PT, 45.9% (45/98) of borderline PT, and only 2.6% (1/39) of malignant PT. This difference was statistically significant (p<0.0001). Pushing borders were observed in 58.3% (194/333) of cases, while infiltrative borders were observed in only 5.4% (18/333) of cases. Stromal atypia, mitosis, intra-tumoral necrosis, and stromal overgrowth were significantly associated with histopathological grades (p<0.05). Malignant PT were more likely to exhibit marked stromal atypia and overgrowth, higher mitotic activity, and intra-tumoral necrosis compared to benign and borderline PT (**Table 1**).

### Recurrence

**Figure 1:**
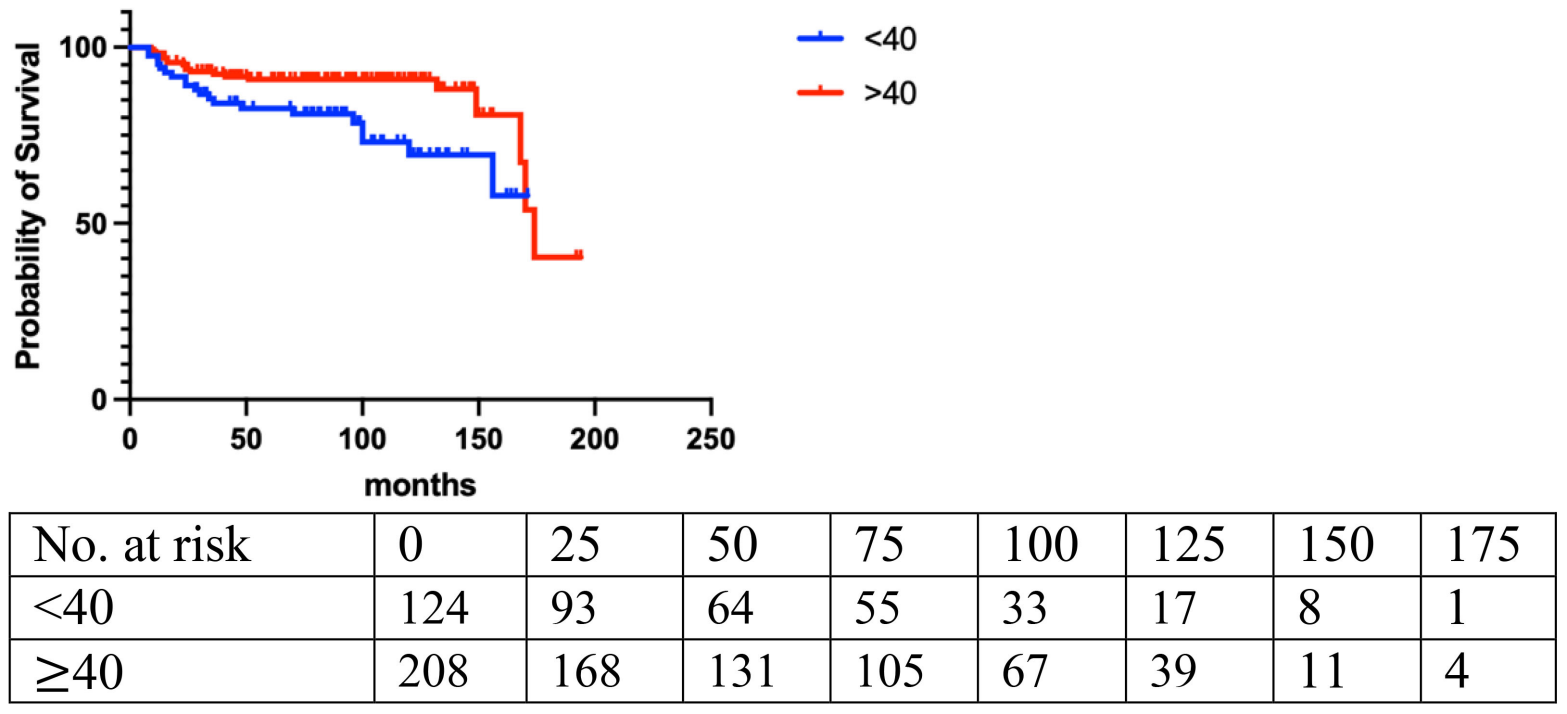
Age (p=0.008).

**Figure 2:**
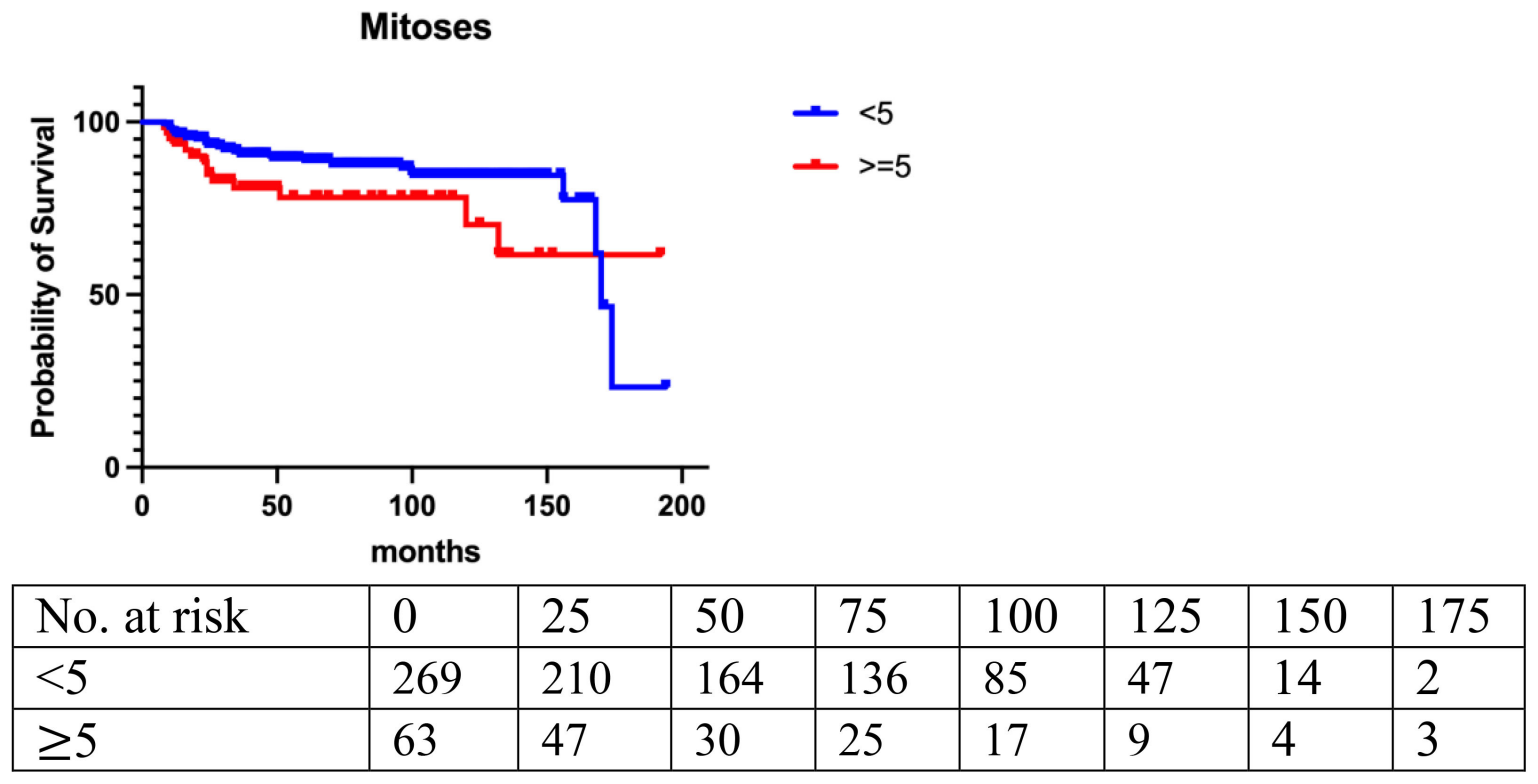
Mitoses (p=0.025).

**Figure 3:**
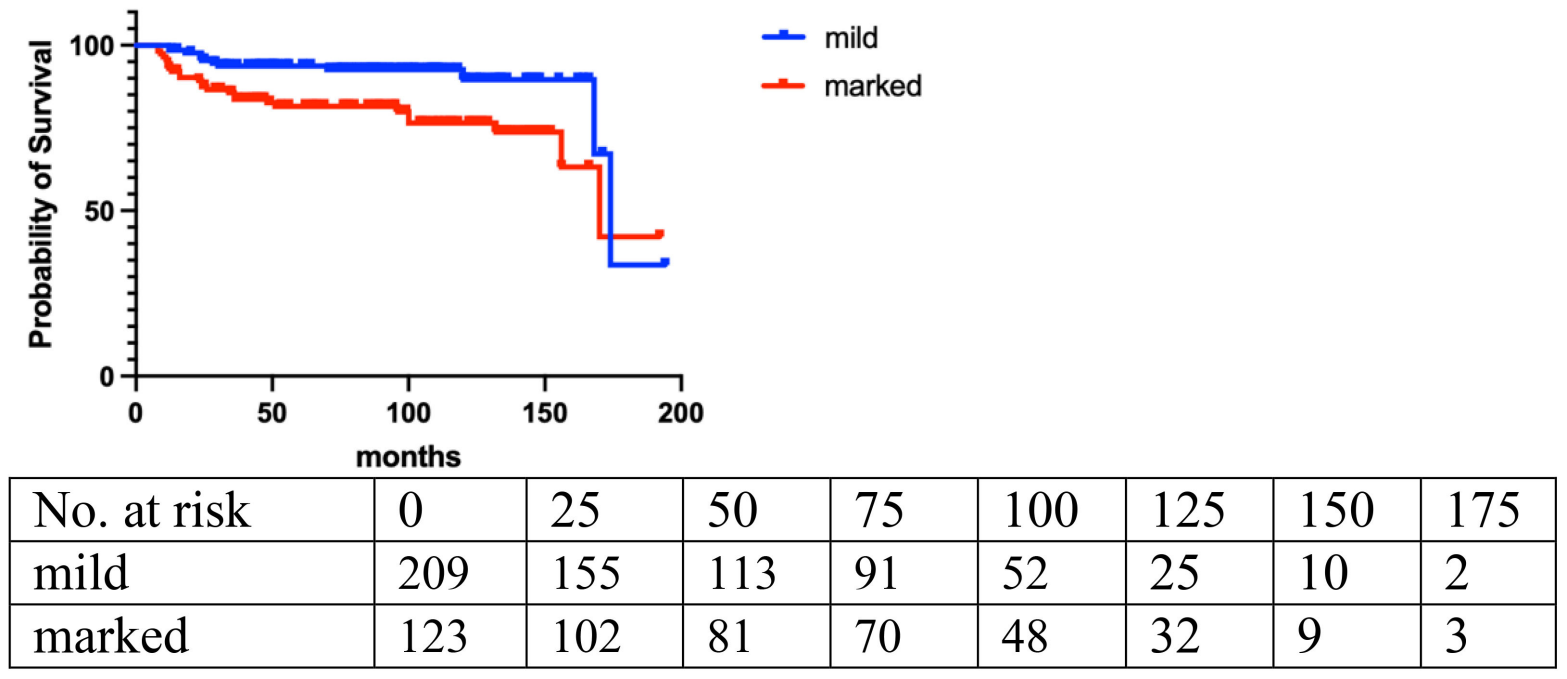
Stromal atypia (p=0.004).

Local recurrence (LR) occurred in 14.7% (n=49) of the entire cohort, with a median follow-up of 42 months. The LR rate was 10.2% (20/196) for benign, 18.4% (18/98) for borderline, and 28.2% (11/39) for malignant PT. LR rates were assessed based on age, tumor size, final closest margin, surgical management, PT grade, and histopathological features (**Table 2**). Logistic regression analysis revealed a significant correlation between LR and age [<40 vs. ≥40: odds ratio (OR)=2.04; 95%CI, 1.13 to 3.68; p=0.008]. Histologically, LR was associated with marked stromal atypia (mild vs. marked, odds ratio [OR] =0.43; 95%CI, 0.25 to 0.77; p=0.0031), stromal overgrowth (yes vs. no, odds ratio [OR] =0.56; 95%CI, 0.32 to 0.98; p=0.01), and active mitosis per 10 high power fields (HPF) (<5 vs. ≥5: OR=0.88; 95%CI, 0.49 to 1.59; p=0.03) (**Figures 1**–**3**).

**Table 2:** Kaplan-Meirer model predicting 5-year DFS.

Predictor	Recurrence	5-year DFS	Chi square	P
Age				
≤40	25	0.82	6.63	0.008
>40	24	0.9		
Tumor size				
≤5cm	31	0.86	0.18	0.67
>5cm	18	0.88		
Final closest margin				
≤1cm	22	0.88	0.39	0.53
>1cm	26	0.85		
Stromal cellularity				
mild	7	0.94	1.73	0.19
moderate/marked	32	42	0.85	
Stromal atypia				
mild	19	0.91	8.75	0.003
moderate/marked	26	30	0.8	
Stromal overgrowth				
yes	19	0.92	4.25	0.04
no	30	0.81		
Mitoses per 10 hpf				
≤5	35	0.89	4.63	0.03
>5	14	0.78		
Phyllodes grade				
Benign	22	0.89	1.57	0.45
Borderline	25	26	0.86	
Malignant	9	9	0.84	
Surgery management				
LE	22	0.87	0.3	0.96
WLE	15	0.87		
BCS	2	0.86		
Mastectomy	9	10	0.88	

**Figure 4:**
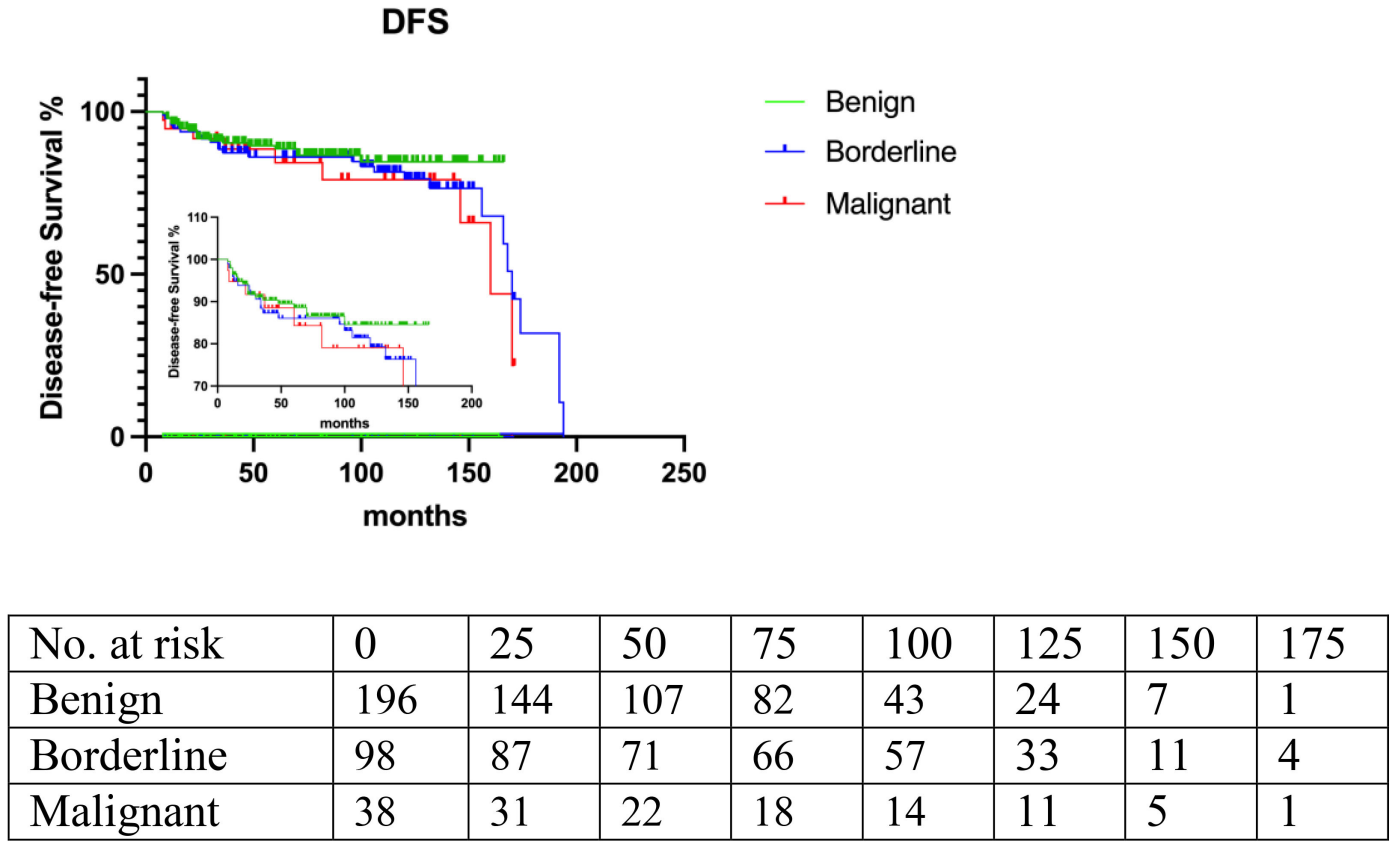
Histopathological grade: benign, borderline, malignant. (p=0.06).

**Figure 5:**
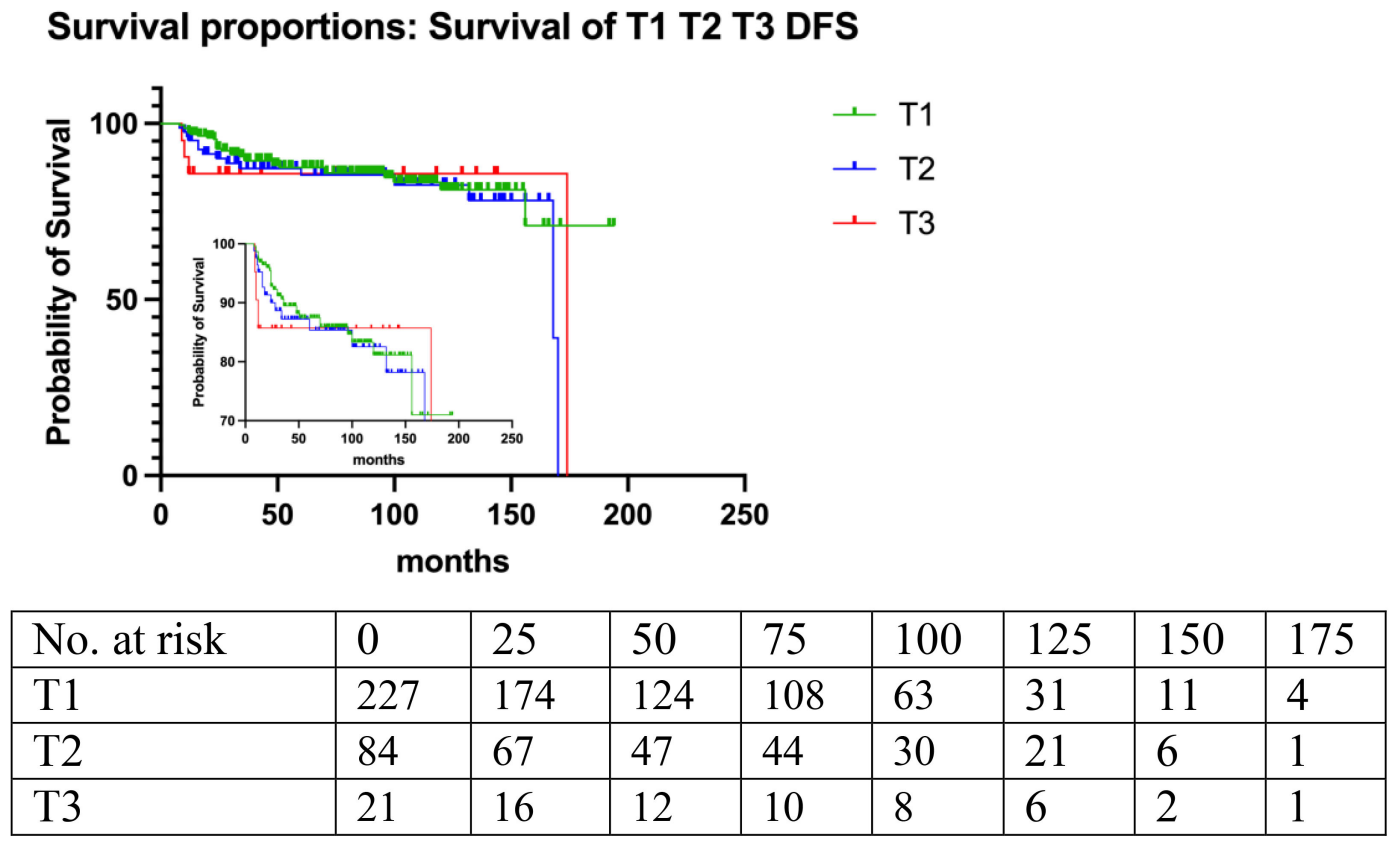
Tumor size (p=0.41).

Logistic regression analysis revealed no significant association between LR and surgical management (LE vs. wide margin: OR=0.89; 95% CI, 0.48 to 1.66; p=0.46), or tumor size (<5cm vs. >5cm: OR=0.88; 95% CI, 0.49 to 1.59; p=0.67). There was no significant association between LR and final margin, surgery management, stromal cellularity, histological border, and stromal overgrowth at diagnosis (**Table 3**, **Figures 4**, **5**). The correlation between LR and various surgical management strategies was analyzed in each subgroup graded by histopathological diagnosis (p=0.09 in benign PT, p=0.3 in borderline PT, p=0.56 in malignant PT), and no significance was identified (**Table 4**).

**Table 3:** Logistic regression models predicting likelihood of local recurrence.

Predictor	LR	OR	95% CI	Chi square	P
Age					
≤40	25	2.04	1.127 to 3.682	6.63	0.01
>40	24				
Tumor size					
≤5cm	31	0.88	0.4904 to 1.591	0.18	0.67
>5cm	18				
Final closest margin					
≤1cm	21	0.84	0.4837 to 1.467	0.3894	0.53
>1cm	28				
Stromal cellularity					
mild	7	0.59	0.3011 to 1.165	3.271	0.07
moderate/marked	32	42			
Stromal atypia					
mild	19	0.43	0.2452 to 0.7668	8.75	0.003
moderate/marked	26	30			
Stromal overgrowth					
yes	19	0.56	0.3207 to 0.9849	5.995	0.01
no	30				
Mitoses per 10 HPF					
<5	35	0.57	0.2803 to 1.179	4.63	0.03
>5	14				
Phyllodes grade					
Benign	22			4.872	0.03
Borderline	25	26			
Malignant	9	9			
Surgery					
LE	22				
WLE	15			0.557	0.46
BCS	2				
Mastectomy	9	10			

**Table 4:** The correlation between LR and surgical managements in subgroups by log-rank test.

		Number	LR	Chi square	P
Benign PT	LE	66	7	2.79	0.09
	WLE	59	6		
	BCS	6	0		
	Mastectomy	7	1		
Borderline PT	LE	30	5	1.08	0.3
	WLE	34	9		
	BCS	8	2		
	Mastectomy	26	3		
Malignant PT	LE	5	2	0.34	0.56
	WLE	11	1		
	BCS	4	0		
	Mastectomy	19	8		

All cases of local recurrence were managed with secondary surgical interventions. Specifically, for benign phyllodes tumors (PTs), 13 recurrent cases underwent local excision with wide margins. In the case of borderline PTs, 2 recurrent cases were treated with local excision and wide margins, and one of these cases received adjuvant radiotherapy (RT). The remaining 17 cases of recurrence were managed with mastectomy. For malignant PTs, 3 cases were treated with mastectomy, and 8 cases underwent local removal of the recurrent tumors. This approach reflects the complexity of managing recurrent PTs, particularly in the context of their variable biological behavior and the need to balance surgical margins with the potential benefits of adjuvant therapy.

### Adjuvant radiotherapy and chemotherapy

A limited number of patients underwent adjuvant radiotherapy (n=3, 0.9%) or chemotherapy (n=2, 0.6%), therefore, the evaluation of the effectiveness of adjuvant therapy was limited.

### OS and DFS

Kaplan-Meier regression analysis showed that the 5-year disease-free survival (DFS) rates were 86.6% for benign PT, 86.3% for borderline PT, and 83.5% for malignant PT according to histological grade. The DFS rates tended to decrease from benign to malignant subtype, although this trend did not reach statistical significance (p=0.06).

**Figure 6:**
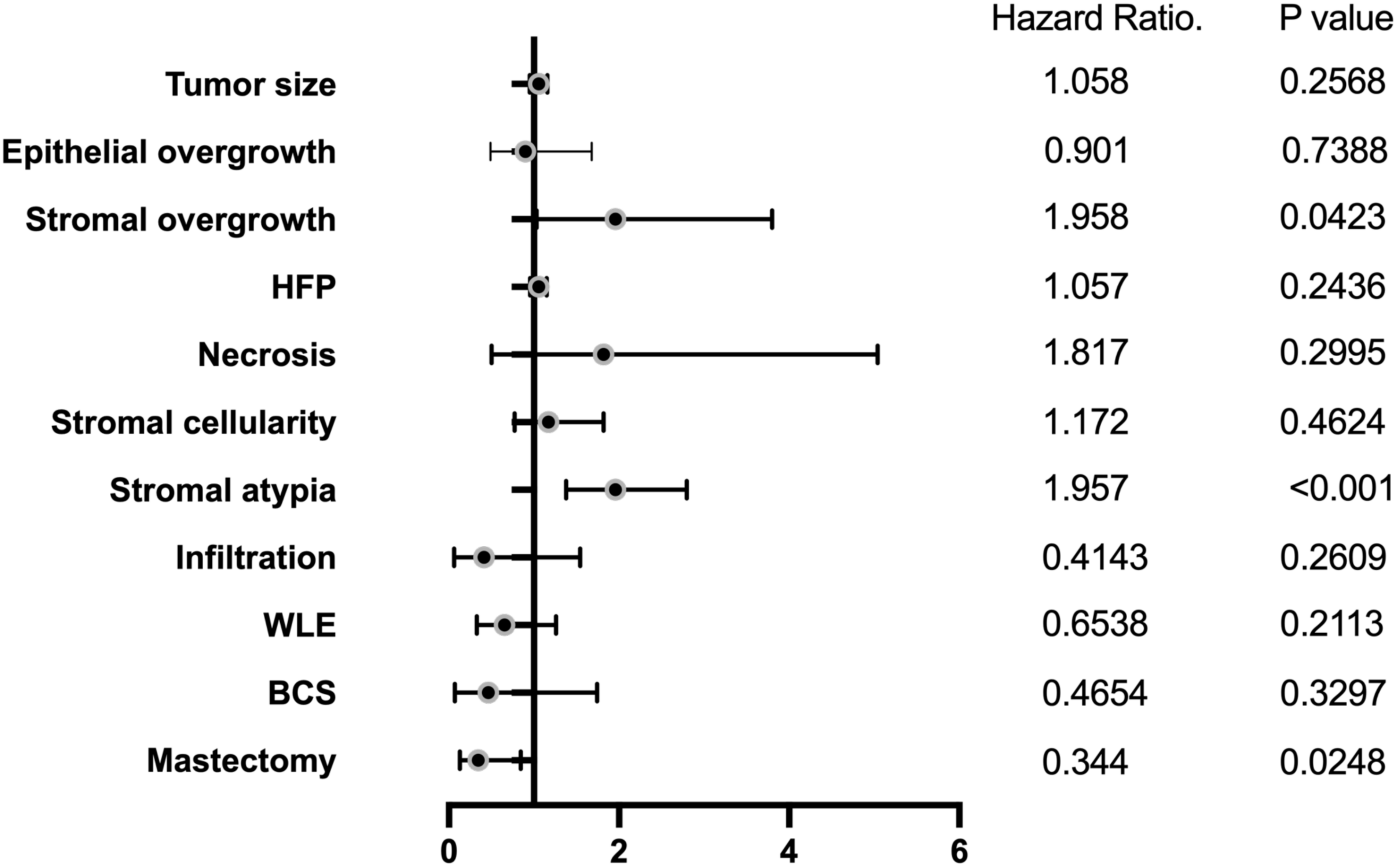
Multiple predictors of local recurrence.

Multivariate COX proportional hazards model analysis revealed that significant risk factors for local recurrence (LR) and DFS included stromal atypia (HR=1.96, 95% CI, 1.38 to 2.798; p<0.001), stromal overgrowth (HR=1.96, 95% CI, 1.03 to 2.8; p<0.05), and mastectomy (HR=0.34, 95% CI, 0.13 to 0.84; p<0.05) (**Table 3**, **Figure 6**).

## Discussion

This study aimed to assess the management and prognosis of patients diagnosed with PT in a single institution over a 13-year period. A total of 333 patients were included in the analysis, and the study evaluated various clinical and histological factors to predict the recurrence rate. Most cases underwent lumpectomy, widely lumpectomy, breast conserving surgery, or mastectomy, despite the NCCN guideline recommending wide margin (>1cm) as the standard management for borderline PT and MPT. Surprisingly, the study found that wide margin was not significantly associated with local recurrence or DFS. Instead, age, stromal overgrowth, stromal atypia, and mitosis were identified as independent risk factors for recurrence, rather than surgical margin.

The diagnosis of PT relies on mammography, ultrasound, MRI and pathology (13). However, these imaging techniques often yield nonspecific results, which can complicate decision-making regarding surgical management (14). In this study, we found that the age of patients and tumor size increased gradually according to the pathological classification of benign, borderline, and malignant PT (p<0.05). Although the concordant diagnostic frequency of PT was found to be about 73.3% in previous reviews by comparing intraoperative frozen sections to permanent histology (15), this dilemma still undermines the value of intraoperative frozen section in the diagnosis of PT, as well as the wide excisional rate of borderline or malignant PT. Moreover, up to 20% of positive margins are identified in postoperative histological examination, which may require additional re-excision according to guidelines (16). The rate of re-excision ranges from 10% to 50% due to variable reasons (17). In our study, most cases of benign PT (93.4%, 183/196) underwent surgical lumpectomy with negative margins due to uncertainty in the rapid frozen section or core needle biopsy (CNB) results. On the other hand, 65.3% (64/98) of borderline PT and 41.0% (16/39) of malignant PT underwent lumpectomy with negative margins. The surgical margin was less than 1 cm in 68.4% of benign PT, 36.7% of borderline PT, and 10.3% of malignant PT, which significantly reduced after comparing with the final histopathology results (p<0.0001). With sufficient tissue for standard and immunological pathology examinations for borderline and malignant PT, we were able to verify the pathologic diagnosis and recommend a second resection with a wider margin in accordance with the NCCN guidelines.

In our study, we found that over half (54.7%) of cases underwent LE, which is contrary to current guidelines. However, only a small percentage of patients were willing to undergo re-excision or mastectomy, with most opting for observation due to uncertain oncologic benefit, low rates of residual PT, and cosmetic concerns (18). Despite this deviation from guidelines, there was no significant difference in local recurrence rates between the surgical treatment cohorts, indicating that recurrence was not a result of surgical management. These findings align with a recent study published in the Journal of Clinical Oncology, which found that wide margins were not an independent risk factor for local recurrence (18). Another study of 246 cases reported that 44% of benign PT underwent re-excision, but only 9% were found to have residual PT in the margins (19). In our study, we found no significant difference in LR rate between patients with benign PT who underwent re-excision and those who underwent observation alone (p=0.7 vs. 0.2). Even all risk factors are considered, there was no difference in the local recurrence of benign PT between open surgery and ultrasound-guided vacuum-assisted biopsy, suggesting that tumor margins may not be a significant factor in predicting recurrence (20). Therefore, there is an urgent need to determine the appropriate management strategies, including surgical margins and prognostic risk factors, for patients with PT.

Benign PT accounts for a significant proportion of PT (60-75%), with a reported lower LR rate of 10-20% when treated with WLE (21). Several investigations have shown no difference in LR between LE and WLE, potentially minimizing the risk of surgical margins (22, 23). Our study found that 11.22% of benign PT recurred, with no significant difference among various surgical management approaches (p=0.09), which is consistent with previous reports. However, the confusing and rapidly frozen pathological outcomes in real-world practice have hindered the WLE rate for borderline PT, which accounts for 10-20% of PT and has been identified with an LR rate of 14-30% by previous studies (24, 25). In a cohort of 90 cases of PT, 52 exhibited positive surgical margins. However, there was no significant difference observed between those who underwent re-excision and those who did not. The current literature provides limited and inconclusive evidence that broad margins in surgical management definitively minimize recurrence (23). In addition, it is noteworthy that the LR rate of borderline PT after WLE has been reported as high as 25% (26). Continuing with our investigation, in our sample, the LR rate of borderline PT was found to be 19.4%. Of the patients, 30.6% underwent lumpectomy (LE), while 34.7% underwent WLE. However, our retrospective investigation did not reveal a significant difference between the four surgical care techniques (p=0.30), thus supporting previous conclusions. For malignant PT cases, surgical management with wide margins or mastectomy was recommended, followed by adjuvant radiotherapy or chemotherapy as necessary (27, 28). It is interesting to note that Spanheimer et al. reported a relatively low LR rate of 12% in 71 patients with borderline or malignant PT, which is comparable to the rate seen in benign PT cases (29). In our study, the LR rate of malignant PT was found to be 28.2%, with most patients undergoing mastectomy (48.7%) or WLE (28.2%) and a median survival time of 160 months. Our results also showed no significant difference in recurrence between different surgical managements or margins, as determined by log-rank testing. However, we acknowledge that a larger, multicenter investigation is needed to confirm the effectiveness of surgical margins in reducing local recurrence in PT cases. Such research could help to guide optimal surgical management and improve patient outcomes.

As surgical margins or different surgical managements were found to be not significant predicting factors of local recurrence (LR) in our retrospective investigation, we further explored other potential factors. Our series revealed a relatively low LR rate of 15.6% (n=52) over a 13-year follow-up period, which is consistent with a recent report of an LR rate of 6.3% during an 8-year follow-up (30). Our analytic results show slightly higher local recurrence (LR) rates compared to a previous large series of 546 cases, which reported a 2.7% LR rate in a 10-year follow-up. In our retrospective study, the 5-year disease-free survival (DFS) rate was found to be 86.6% for benign PT, 86.3% for borderline PT, and 83.5% for malignant PT, but the decline was not statistically significant (p=0.06). The 5-year DFS rates for subgroups classified by margins (<1cm and ≥1cm) were relatively high at 87.8% and 86.6%, respectively, with no significant difference observed (p=0.70), consistent with recent reports. One such study reported a 5-year DFS rate of 87.8% for margin-negative patients and 85.1% for margin-involved patients, concluding that margin status was not significantly associated with recurrence (31). Thind et al. conducted a meta-analysis of 10 retrospective studies based on MEDLINE and Embase (1990 to 2019) to investigate whether margins <1cm are sufficient for excision to prevent local recurrence (LR) in PT cases. Their study was conducted in accordance with the Preferred Reporting Items for Systematic Reviews and Meta-Analyses (PRISMA) guidelines (32). The meta-analysis concluded that margins <1cm may provide adequate excision to prevent LR in PT cases. In another meta-analysis, it was concluded that different surgical management strategies with margins <1cm were not significantly associated with the risk of local recurrence (LR) in all grades of PT. The meta-analysis systematically analyzed 34 articles with Newcastle-Ottawa Scale (NOS) scores above 5 using RevMan5.3 software (33). These findings support the importance of individualized surgical management plans for PT cases based on the tumor’s characteristics and patient factors. Numerous large series around the world demonstrated that positive margins were not significantly associated with LR, including Bedi D. (N=270) (34), Belkacemi et al. (N=443) (35), Cheng et al. (N=182) (36), Tan et al. (N=605) (37), and Co et al. (N=465) (38); Increasing data has shown that negative margins are not necessarily relevant to LR and disease-free survival (DFS). However, the margins issue remains challenging to draw a conclusion due to the lack of supporting data. The rarity of PT and the limited availability of high-quality data from multiple institutions and prospective studies makes it difficult to draw definitive conclusions about the optimal management of surgical margins.

In our investigation, we examined several clinical and histological parameters, including age, tumor size, surgical margins, surgical procedure, stromal atypia, stromal overgrowth, and mitoses, to explore the underlying predicting factors of LR. Our results showed that age, stromal overgrowth, stromal atypia, and mitoses were significant risk factors for LR, as identified by logistic regression analysis. Specifically, age (<40 vs. ≥40), stromal atypia (mild vs. marked), stromal overgrowth (yes vs. no), and mitoses (<5 vs. ≥5) were found to be independent prognostic indicators for LR by log-rank regression analysis. Interestingly, our analysis showed that tumor size and surgical managements were not associated with DFS. However, other studies have reported different findings. For instance, Olaya J reported a LR rate of 14.8% with high-grade and mastectomy as high-risk factors, while a single center covering 192 cases revealed LR of 16.1% and distant metastasis of 6.3% in the 10-year follow-up (39). We came to the conclusion that tumor size, hemorrhage and margin status were independent predictors of LR and OS (40). A Canadian retrospective study examining 150 PT found that the overall LR was 7.3%, with 45% of benign, 27% of borderline, and 27% of malignant lesions (41). It is important to note that the need for wide margins to reduce LR varies based on the histopathologic type of PT. Borderline and malignant PT require a wide margin to reduce the risk of LR, while benign PT has no significance regarding margins. Recent research has also shown that negative margins may not be significant when LR reduction surgery is performed, particularly in the case of benign PT.

The NCCN guidelines suggest that radiotherapy should be considered for malignant PT when the risk of LR is high, which occurs in around 10-15% of cases (42, 43). Several studies have recommended adjuvant radiation for malignant PT to reduce the risk of LR, but its effect on overall survival has been found to be minimal (44, 45). A multivariate study of 1353 MPT patients with a follow-up of up to 331 months using Kaplan-Meier curves and Cox proportional hazards analysis found that adjuvant radiation did not improve overall survival (46). Another retrospective study of 108 PT patients with a follow-up of 56 months found no significant difference in the LR rate between borderline and malignant PT patients who did or did not receive adjuvant radiotherapy (47). The 5-year overall survival rates were 52% and 45% in the radiation and non-radiation groups, respectively (p=0.54). In our series, only 8.6% and 2.9% of malignant PT cases underwent adjuvant radiotherapy and chemotherapy, respectively. Adjuvant radiotherapy is not recommended for benign PT patients to prevent LR due to the potential risk of radiation-induced second malignancy (48). Therefore, adjuvant radiotherapy is controversial and supported mainly for large and high-grade phyllodes tumors (2, 35, 49).

There are several limitations to our study, which was a single-center and retrospective analysis, potentially introducing bias. The clinical characteristics of patients with benign, borderline, and malignant PT were not well-balanced, which may have impacted the statistical analyses. Additionally, larger studies are needed to further explore optimal surgical margins and prognostic indicators for PT.

Despite these limitations, our study contributes to the long-term prognosis of PT by providing a relatively long median follow-up period. Our findings suggest that wider surgical margins may not be necessary for all types of PT, as we found no significant difference in LR rate or DFS among different surgical managements. We also identified age, stromal overgrowth, stromal atypia, and mitotic rate as important risk factors for prognosis using multivariate COX proportional hazards analysis.

## Data Availability

The original contributions presented in the study are included in the article/supplementary material. Further inquiries can be directed to the corresponding author.
